# Ultra-processed foods and type 2 diabetes: more fundamental research is needed

**DOI:** 10.1016/j.lanepe.2024.101084

**Published:** 2024-09-19

**Authors:** Joseph A.M.J.L. Janssen

**Affiliations:** Department of Internal Medicine, Erasmus MC, Dr Molewaterplein 40, 3015 GD, Rotterdam, the Netherlands

Incidence of type 2 diabetes is worldwide progressively increasing. It is generally assumed that this increase is due to changes in environmental rather than genetic factors. Diets and food supplies are increasingly based on ultra-processed foods (UPFs).^1^ UPFs are defined as industrially manufactured, ready-to-eat/heat formulations that are largely devoid of whole foods. Although UPFs form are highly heterogeneous food category, many UPFs are high in energy density, sugars, trans fats as well as additives. At present UPFs are responsible for more than 50% of daily total energy intake in high income countries.^2^ A systematic review of 7 prospective cohort studies reported a pooled relative risk (RR) of 1.50 (95% CI: 1.18, 1.90) for type 2 diabetes when comparing participants with the highest total UPF intake versus participants with the lowest total UPF intake.^3^ In a meta-analysis, the same authors found that each 10% increment in total UPF intake was associated with a 12% higher risk on type 2 diabetes, suggesting a linear dose response association.^3^

In this issue of *The Lancet Regional Health–Europe*, Dicken et al. report results of a large prospective analysis within the European Prospective Investigation into Cancer and Nutrition (EPIC) cohort.^4^ Dietary intake was assessed at baseline using dietary questionnaires and classified according to the Nova classification.^4^ Over an average 10.9 years follow-up of 311,892 individuals, 14,236 people with type 2 diabetes were identified. In line with previous studies, they found that each 10% increment of total daily food intake from UPF (%g/day) was associated with 17% (95% confidence interval (95% CI): 1.14–1.19) higher incident type 2 diabetes.^4^ The authors used several models to calculate potential modifying effects of replacing UPFs by unprocessed/minimally processed food and processed culinary ingredients or proceeds foods.^4^ When replacing UPFs by unprocessed/minimally processed food and processed culinary ingredients or processed foods, they found (in their theoretical model) a lower incidence of type 2 diabetes.^4^ They therefore suggested that replacing intake of specific UPF should be recommended to reduce lowering type 2 diabetes risk.^4^

However, an important yet unanswered question remains how UPFs contribute to type 2 diabetes. It has been speculated that increased type 2 diabetes incidence may be because UPFs contain high(er) amounts of fat and sugar than processed food, both of which are considered contributors to type 2 diabetes and adiposity. Experimental evidence indicates a robust causal relation between UPFs and increased energy, carbohydrate and fat intake: 14 days ad libitum intake of UPFs caused increased body weight (+ 0.9 kg), energy intake (+ 508 kcal/day), carbohydrate intake (+280 kcal/day) and fat intake (+230 kcal/day) compared to an unprocessed diet despite the fact that both diets were matched for the amount of calories, sugar, fat, sodium, fiber, and macronutrients.^5^ Excess nutrient ingestion may stimulate insulin secretion, fat storage, and consequent type 2 diabetes.^6^ Increased adiposity mediated by (high consumption of) UPFs would fit within the Personal Fat Threshold (PFT) hypothesis, which postulates that each individual has a PFT which, if exceeded, makes the development of type 2 diabetes likely: when an individual (of any BMI) can no longer store triglyceride safely in subcutaneous fat depots: this stimulates excess fat deposition in the liver, excess hepatic fat export and exposure of pancreatic β cells to excess lipid, and this promotes type 2 diabetes.^7^ Thus, UPFs could contribute to an earlier exceeding of the PFT and thereby to type 2 diabetes.

In addition, many UPFs contain food additives, which may also potentially influence type 2 diabetes risk. For instance, carrageenan, a thickening and stabilizing agent often used as additive in many UPFs, might contribute to the development of type 2 diabetes by impairing glucose tolerance, increasing insulin resistance, and inhibiting insulin signaling.^8^ However, for most additives present in our food, human data on long-term health impacts are still lacking. Moreover, UPFs may be contaminated by chemicals such as bisphenols and phthalates, which are commonly used in food plastic packaging.^8^ Exposure to bisphenol-A (BPA) may augment diabetes risk by affecting pancreatic β-cell function and/or insulin action.^9^

It should be underscored that at present a clear causality between exposure to UPFs and the development of type 2 diabetes has not definitively been established. Moreover, the intake of some UPFs might also decrease type 2 diabetes risk. For example, intake of cereals, dark and whole-grain breads, packaged sweet snacks, and yogurt have all been associated with lower type 2 diabetes risk.^10^

The scientific evidence regarding the role of UPF in the pathogenesis of type 2 diabetes is insufficient to propose changes in evidence-based guidelines and health policies in the management of type 2 diabetes. A better understanding of how UPFs, additives, and chemicals in UPF, are causally linked to type 2 diabetes requires urgently more fundamental research.

## Declaration of interests

I declare no conflict of interest.
